# Navigating the maze of active ageing measurement: untangling methodological and theoretical issues in the UJACAS questionnaire

**DOI:** 10.1007/s40520-025-02953-5

**Published:** 2025-03-13

**Authors:** Andrea Bosco, Antonella Lopez

**Affiliations:** 1 Department of Educational Sciences, Psychology, Communication, University of Bari, Aldo Moro, Bari, Italy; 2 Department of Humanities, Social Sciences and Education, University of Molise, Campobasso, Italy

**Keywords:** Active Ageing, Measurement Ttool, Psychometrics

## Abstract

Active Ageing (AA) involves maintaining engagement in physical, social, and mental activities in older adulthood, reflecting societal values and political agendas. Despite various measurement tools, there is no universal standard. The University of Jyväskylä Active Ageing Scale (UJACAS) is increasingly used in Europe but faces inconsistencies from varying response scales across versions. This perspective paper examines these discrepancies and proposes a refined model for AA measurement, emphasizing the dimensions of motivation, ability, opportunity, and activity frequency. The goal is to improve the accuracy of AA assessments by adjusting for personal and environmental limitations, ensuring a fair evaluation that captures the complexities of ageing. The proposed adjustments to the UJACAS scoring model include recalibrating the equation to account for personal and environmental limitations, integrating motivation as a separate measure, and exploring adaptive methodologies to ensure fairer and more nuanced assessments of Active Ageing, particularly for individuals facing disabilities or obstacles. By addressing these issues, this perspective paper aims to enhance the effectiveness of AA tools for older adults. Moreover, adopting these methodological challenges has significant societal implications. Precise measurement of AA is essential for tailoring interventions to the needs of diverse older populations and for designing policies that promote inclusive, healthy and safe ageing. Enhancing the accuracy of AA assessments can inform strategies that empower older adults, mitigate disparities, and foster resilience in ageing communities. This study highlights the necessity of advancing AA measurement frameworks to support evidence-based decisions in public health and social policy.

**Supplementary Information:**

The online version contains supplementary material available at 10.1007/s40520-025-02953-5.

## Introduction

Active Ageing (AA) reflects the ability to maintain physical, social, and mental engagement throughout older adulthood. It goes beyond mere health, it encompasses participation in meaningful activities, maintaining autonomy, and adapting to changing capacities [1].

The concept, which emerged in the 1990s through initiatives by the WHO and various governmental and non-governmental organizations, provides a policy framework highlighting the interconnectedness of activity, health, independence, and successful ageing. More recently, The World Health Organization (WHO) defines active ageing as “…*the process of optimizing opportunities for health*,* participation*,* and security in order to enhance quality of life as people age*” [2].

AA is not just a public health or social issue, but a concept highly valued in politics shaped by economic, social, and ethical considerations. How societies define and support AA reflects their values around productivity, equity, and care, making it a deeply political agenda. Developing a system of shared indicators based on equally shared reference models in AA is crucial for shaping global public health policies aimed at enhancing the quality of life for older adults and addressing the challenges associated with ageing. Notwithstanding the importance of a tool to measure and monitor active ageing, disagreements remain among experts and institutions regarding a clear operational definition of active ageing and the most appropriate tools for the assessment [3].

Cultural, demographic, and policy differences significantly influence how AA is measured, as these factors shape the values, priorities, and frameworks used to define and assess ageing. Cultural differences determine how older adults are perceived in society, with some cultures emphasizing family and community support, while others focus on individual independence and productivity [4]. Demographic factors, such as age distribution, urbanization, and socioeconomic status, affect the types of activities and support structures available to older adults, thereby influencing the tools used to measure AA [3]. Additionally, policy variations across countries reflect different approaches to healthcare, social welfare, and inclusion, which can affect the design and implementation of AA measurement tools to ensure they are relevant and applicable in specific contexts [3]. These differences highlight the need for adaptable, standardized AA measurement frameworks that respect cultural and contextual variations while maintaining comparability across regions. Furthermore, addressing global ageing challenges requires harmonized, inclusive assessment frameworks that respect cultural nuances while supporting cross-national research.

Although numerous tools for measuring active ageing have been developed, their conception within various cultural contexts across different countries prevents the establishment of a single - shared - gold standard. This diversity adds to the uncertainty faced by researchers and healthcare professionals when choosing the most appropriate tool for assessment. As emerge from the scoping review of Xiao and colleagues [3] eight of the 22 reported studies focused on assessing active ageing at the macro level (e.g. policies, strategies, and societal initiatives), while four explored it at the individual level. Thirteen studies were based on the World Health Organization’s theoretical framework for active ageing [5], whereas the other nine relied on alternative theoretical models. Furthermore, only two active ageing assessment tools in the literature were specifically designed for subgroups of older adults, addressing factors such as gender and age. The authors suggest that future efforts should focus on developing individual-level assessments and creating measurement tools tailored to the needs of diverse demographic groups.

The aim of the present perspective study is to focus on the assessment tool for AA that currently has the highest number of official versions in Europe, highlighting the points of imperfect convergence between the different versions. A second objective is to propose our own representation of the AA measurement model based on the University of Jyväskylä Active Ageing Scale (UJACAS) [6], and to suggest how to calculate the final Active Ageing score. Despite its growing adoption, UJACAS faces challenges stemming from discrepancies across national versions, particularly in response scales and scoring models. These inconsistencies hinder the comparability of research findings and limit the tool’s utility in cross-national studies. A lack of standardization compromises the reliability of AA assessments and the ability to derive actionable insights from international datasets. Addressing this issue is essential to ensure the scale’s validity and facilitate robust research that can inform effective interventions and policy development worldwide.

## Active Ageing: conceptual framework and measurement challenges

It is well known the interplay between biological and psychosocial factors in ageing [7]. The integrated life course model of ageing [8] combines various factors that influence ageing, including physiological capacity (such as central nervous system integrity, endocrine and immune system balance, and age-related changes in body composition, energy capacity, and consumption), emotional health, reproductive health, and environmental factors. These environmental factors encompass physical elements (e.g., material conditions, pollutants, and assistive technologies) and social aspects (e.g., family, neighbourhoods, retirement, and healthcare). The model also emphasizes the interaction of these factors across specific life course phases, including conception, prenatal, prepubertal, pubertal, maturity, and senescence. It is also known that during ageing replication stress and telomere dysfunction are key biological factors that influence the ageing process, with implications for AA measurement. Telomere length, a biological marker of cellular ageing, reflects the cumulative impact of replication stress, environmental factors, and lifestyle choices on cellular function. Shortened telomeres are associated with reduced cellular regeneration and increased susceptibility to age-related diseases, offering valuable insights into an individual’s biological age [9]. Additionally, epigenetic changes, which affect gene expression without altering the DNA sequence, can provide complementary data on how environmental and psychosocial factors influence biological ageing [10]. Integrating these biological markers into AA measurement bridges psychosocial and biological perspectives, enhancing the accuracy and depth of assessments by considering both the external, behavioural factors and internal, cellular mechanisms that contribute to active ageing. This holistic approach offers a more comprehensive understanding of ageing, addressing both the physical and psychological dimensions of older adults’ lives.

University of Jyväskylä Active Ageing Scale was designed to measure a multidimensional view of AA, as is the case with other similar tools, focusing exclusively on behavioural / psychological variables such as: self-reported goals, abilities, autonomy, and actual activity over the past four weeks. These subscales aim to capture the complexity of active ageing by assessing, through a set of 17 items, the volume of acts but also the individual’s desires, abilities, and opportunities to engage in various activities. Following the original paper, the *will to act* should get across the individual’s motivation and desire to engage in several activities. It reflects the personal goals or aspirations an older adult has regarding their daily occupations. The *ability to act* evaluates the perceived ability or capacity of the person to perform activities. It measures how capable individuals feel they are to engage in the activities, their state of health and functional capacity. The *possibility to act* focuses on the opportunities to be engaged in desired activities. It reflects the extent to which individuals feel to have the autonomy and external circumstances (environmental - in space and time - social, or situational constrains) to pursue those activities. The *frequency /amount of doing activities* measures the actual participation in activities. A five-point Likert-type scale was used, with response options tailored to each item and scored from 0 (indicating the lowest level, such as least active) to 4 (indicating the highest level, such as most active). The scores for each dimension were calculated by summing the individual item scores to form subscales, and the total score was derived by adding together the subscale scores for the entire questionnaire. The total score ranges from 0 to 272, with higher scores reflecting a higher level of AA.

The scale is beginning to see substantial use in Europe, as it has been validated in other three countries. However, notable discrepancies arise in its validation process. While the original Finnish/English versions of the UJACAS and the Turkish validation [11] utilized a 0–4 response scale (from 0, least active, to 4, most active), later adaptations - particularly the German and Swedish versions [6, 12] - employed a 1–5 scale, introducing the risk of inconsistencies in interpretation. These differences may significantly impact data consistency, interpretation, and the comparability of research findings. Moreover, for the inattentive reader, the variation in response scales undermines this standardization, potentially affecting the scale’s reliability and validity across contexts. Such variations highlight the need for standardized adaptation protocols designed to systematically and uniformly adapt a tool, test, or program to a new context, population, or condition.

Noticing these discrepancies prompted us to reflect on how the dimensions of the scale are evaluated and their contribution to measuring AA. This consideration led to rethink how each dimension—will, ability, possibility, and (actual frequency of) activity — plays a role in capturing different aspects of active ageing and also these dimensions are integrated into a comprehensive understanding of an individual’s active engagement. For instance, cultural differences can significantly influence how respondents interpret key concepts such as will and possibility in AA assessments, potentially impacting AA scores. In some cultures, possibility may be understood primarily in terms of access to resources, such as healthcare, social services, and community engagement, which can vary widely depending on local policies and societal structures. In other cultures, it might focus more on social roles and family support systems, which can shape the availability of activities and participation. Similarly, will may be viewed differently across cultures: in some cultures, intrinsic motivation, such as personal satisfaction and well-being, might be emphasized, while in others, extrinsic factors like social expectations, family approval, or community involvement may play a more significant role. These varying interpretations can affect how individuals score on AA assessments, underscoring the need for culturally sensitive tools that account for these differences while ensuring comparability across diverse populations. By examining the interaction of these dimensions more closely, we can ensure they accurately capture the overall measure of AA. First, we are fully convinced with one principle: measuring activity alone may not capture the full picture of AA. The structure of UJACAS aligns properly with this principle. Secondly, all available versions use a linear additive model based on the four evaluated dimensions: people with higher motivation to be engaged, greater abilities (e.g., fewer physical impediments), more opportunities (e.g., fewer environmental barriers to overcome), and a greater volume of activities receive the highest scores of AA. Does the measurement of active ageing work well in this way?

### Will to act

Will to act, motivation, is the drive to engage in activities because they bring pleasure, well-being, and enjoyment, as highlighted by Deci and Ryan’s Self-Determination Theory [13]. Motivation is crucial for evaluating the effectiveness of interventions and adapting activities to individual conditions. While motivation is a key factor in initiating and sustaining activities, practical barriers, such as distance and accessibility, can significantly limit engagement. Even when someone is highly motivated, the physical environment—such as the location of an activity—can be a decisive factor in determining how often they participate [14]. It is our opinion that motivation to engage in activities should still be assessed in order to use it as a proxy for proposing daily activities that are adapted to the participant’s circumstances and residual resources, and that are as similar as possible to those activities that receive the highest consensus in terms of willingness to initiate and maintain the activity (in the case of physical activity [15]). This underscores the importance of addressing both intrinsic motivators (e.g., enjoyment, health benefits) and extrinsic factors (e.g., accessibility, location) when designing programs for older adults. More specifically, intrinsic motivation refers to engaging in activities for their inherent enjoyment or personal satisfaction, whereas extrinsic motivation is driven by external rewards or social influences, such as family expectations or community involvement. Both forms of motivation play essential roles in determining the extent to which older adults engage in activities. Intrinsic motivation often leads to more sustained and meaningful participation, while extrinsic motivation can help initiate and maintain activity, particularly when external factors (e.g., social support or structured programs) are provided. Motivation can also serve as a buffer against age-related declines, particularly in individuals experiencing frailty or facing significant environmental barriers. Those who maintain a high level of motivation, even in the presence of physical limitations or difficult circumstances, often demonstrate greater resilience in overcoming these obstacles [16]. For individuals with high frailty, motivation to act may drive them to pursue activities that support physical and cognitive health, helping to mitigate the negative effects of ageing. By focusing on both intrinsic and extrinsic motivators, interventions can be better designed to support older adults, particularly those with frailty, in maintaining an active and engaged lifestyle despite challenges. For example, interventions that bring activities closer to home or provide transportation services can mitigate these barriers and help translate motivation into actual participation. Creating environments that are conducive to regular engagement can empower older adults to maintain active lifestyles and enjoy the associated physical and mental health benefits.

### Ability and opportunity to act

On these matters, we are convinced that the current way to consider ability and opportunity in the UJACAS needs to be revised. People still manage to participate in activities despite personal difficulties of both physical and mental nature or environmental barriers should receive a significantly higher estimated active ageing score compared to those who, despite engaging in the same number of activities, do not face such challenges. For instance, in urban settings, access to activities may be facilitated by proximity to healthcare services, social spaces, and transport options, while in rural areas, individuals might face greater distance and limited options, creating structural barriers that reduce opportunities for engagement [17]. Chronic health conditions, such as cardiovascular diseases or arthritis, may limit the ability to participate in physical activities, thereby influencing the selection of activities that are less physically demanding. These barriers must be considered when assessing AA, as they significantly impact an individual’s engagement and activity levels. This stems from the principle that overcoming physical or situational barriers requires additional effort and persistence. These individuals often display greater resilience and adaptability, which should be recognized in their AA evaluation [18]. Ability and opportunity are closely linked to concepts of resilience and intrinsic capacity, as individuals often adapt to limitations using psychological and social resources. Resilience enables individuals to maintain activity levels despite challenges, as seen in those with chronic illnesses who modify their daily routines or seek alternative methods of engagement, such as online exercise programs or support groups [19]. The intrinsic capacity to cope with adversity can buffer against declines, enabling individuals to overcome personal or environmental barriers by leveraging internal resources, such as mental toughness, motivation, or social support networks. To accurately assess AA, the dimensions of opportunity and difficulty should be viewed as moderators / mediators of AA. The idea is that these factors externally contributed to modify the levels of AA rather than being intrinsic to it. This perspective aligns with findings from studies on disability and participation [20, 21], which suggest that activity levels should be adjusted based on the challenges faced. The notion of adjusting scores based on the level of impairment is supported by the concept of *disability-adjusted life years* (DALYs), which considers both years lived with disability and years lost due to early death [22]. Similarly, in AA assessments, the degree of impairment should be factored into the scoring equation. The greater the impairment, the higher the adjusted AA score should be, reflecting the increased effort required to participate in activities. Conversely, individuals without impairments might have their scores adjusted downward if they engage in activities with less relative difficulty. Thus, frequency of activity should be *weighted* by the severity of these impediments.

Furthermore, adjusting AA scores for barriers aligns with inclusive policy principles, such as universal design and equity. By considering the impact of environmental and personal limitations, policies can be better designed to ensure that all individuals, regardless of their conditions, have equal opportunities to maintain active and engaged lives. For instance, universal design principles in urban planning can reduce structural barriers, while targeted health interventions for those with chronic conditions can improve their ability to participate in activities. Such adjustments support the creation of inclusive environments where older adults, especially those with impairments or environmental challenges, can remain active and independent [23].

### The UJACAS AA score equation

Currently the equation used to obtain the AA score is the following:

$$\begin{gathered}\:AA\:score = Will\:to\:act + Ability\:to\:act \hfill \\\,\,\,\,\,\,\,\,\,\,\,\,\,\,\,\,\,\,\,\,\, + Opportunity\:to\:act + Frequency\:of\:Activity\: \hfill \\ \end{gathered} $$

The UJACAS questionnaire is one of the most promising tools for gathering valuable information on the level of active ageing, both in research contexts and in primary care and social services for ageing, due to the comprehensiveness of the information it enables to be collected. Notwithstanding this, there should be a reconsideration of the relationship between the measurement of activity frequency, the measurement of willingness/motivation for activities, and the two dimensions of personal and environmental/external limitations. In the first case, we believe that a measure of willingness to act should be used to support personalized interventions aimed at increasing the frequency of preferred activities for which the individual feels they cannot engage as much as they would like. Moreover, personal and environmental limitations should be used to adjust the activity frequency score. We are convinced that both ability and opportunity to act are critical to participation, their impact may differ depending on the specific context, individual circumstances, and the nature of the activity itself. For example, if an individual has limited ability, their participation may be severely constrained, even if they have ample opportunity. Hence, ability is often given a higher weight, as it can be a primary determinant of whether or not an individual can engage in the activity, regardless of external opportunities. While opportunity is important, it could be considered secondary to the individual’s ability to perform the activity. For instance, if an individual lacks the physical capacity to engage in an activity (e.g., due to frailty or disability), having an abundance of opportunities may not significantly alter their ability to participate.

Moreover, regarding the score assigned to, some clarifications are necessary. In our view, the value zero, referring to frequency, ability, or opportunity to act, constrains the frequency score for that particular activity to zero. For instance, a participant reports completing an activity zero times but also indicates a full ability (4) and full opportunity (4) to perform that activity. Under the standard formula, which aggregates these scores, the outcome is a paradoxical contribution of 8 to the total score, despite the activity being reported as not performed. This demonstrates why an activity score of zero must nullify ability and opportunity scores. In the original formula, will to act was also taken into account. The formula with the addition of will to act (4), could produce an item score as high as 12, a potentially misleading outcome for an activity explicitly declared to be unperformed.

Similarly, complete inability to perform an activity, even with assistance, should logically correspond to a frequency score of zero. Furthermore, a zero-opportunity score, which signifies the impossibility of performing the activity, cannot logically yield a non-zero result. These two points clarify why, in our view, any zero-value reported by a participant must result in a sequence of zero values for the corresponding item. The first example illustrates the paradox of achieving a high activity score for an activity reported as unperformed. The second example explains why any non-zero result, in the presence of zero scores for ability or opportunity to act, could be misleading.

Together, these considerations necessitate constraining the formula to handle zero values from weighted computations.

A way to proceed in relation to what has been discussed so far would be to consider the following equation:

$${X_C} = \left\{ {\begin{array}{*{20}{l}}0&{({X_{O\:}} = 0)\: \vee \:({Y_{O\:}} = 0) \vee \:\:({Z_{O\:}} = 0)} \\ {{X_{O\:}} - min(\Delta \:,{\text{max}}(0,\:({X_{max\:}} - \:{X_{\:min\:)}}\:.\:\frac{{{\text{exp}}\left( {{w_{y\:}}.\left( {{Y_{O\:}} - {X_{O\:}}} \right) + {w_{Z\:}}.\left( {{Z_{O\:}} - {X_{O\:}}} \right)} \right) - 1}}{{{\text{exp}}\left( k \right) - 1}}}&{otherwise.} \end{array}} \right.$$

where X_0_ is the original score of frequency of activity; X_max_ and X_min_ are the upper and lower limits of the Likert scales (i.e., 4 and 1, zero is excluded because, for this value, regardless of which of the three variables it appears in, it receives treatment that is not accounted for in the general equation); w_y_ and w_z_ are the weights for the variables Y_0_ (ability to act) and Z_0_ (opportunity to act); k is the *scaling constant*, which can be calculated as the weighted maximum deviation; Δ is the maximum allowed variation.

For the reasons outlined above, the logical condition ensures that if any of the three variables equals zero, the corrected score is set to zero. From a purely logical standpoint, lacking personal resources (physical, mental) and/or lacking opportunities due to environmental limitations (time, social, economic) that cannot be overcome even with assistance, makes any frequency value for the activity greater than zero inapplicable. This reflects the absence of prerequisite contributions and maintains consistency in the scoring process. Otherwise, if none of the conditions are true (all variables are non-zero), the correction is applied.

The adjustment can be explained in the following steps:

1 $$\:{w}_{y\:}.\left({Y}_{O\:}-{X}_{O\:}\right)+{w}_{Z\:}.\left({Z}_{O\:}-{X}_{O\:}\right)$$

The adjustment is calculated using an exponential function, which reflects the influence of ability to act (Y_0_) and opportunity to act (Z_0_) on the score of the activity frequency. The exponential model ensures that small differences between the observed score and the limiting variables result in proportionally smaller corrections, while larger differences produce more significant adjustments. This is formulated as: were w_y_ and w_z_ are the weights for the variables Y_0_ (ability to act) and Z_0_ (opportunity to act) and X_0_ is the original score of frequency of activity. While this approach may seem counterintuitive — using the original score of frequency both as a starting point and as a reference for correction — it is essential for capturing the discrepancy between the score and the prerequisite variables. Rather than disregarding the original value, this method leverages it to ensure the correction aligns with the contextual factors. This deviation determines both the direction (increase or decrease) and proportionality of the correction, ensuring consistency with the extent of the discrepancy. Different weights can be attributed to the variables. The different weights reflect the degree of importance assigned to ability to act and opportunity to act in promoting or constrain the frequency of activity. Indeed, there are reasons to assume that the weight of abilities (or conversely, personal constraints) should be considered greater than the weight of environmental constraints [24–25]. Since the weights of the variables must equal 1 to ensure a balanced distribution of influence among the factors, and because of this normalization avoids inflating or diminishing the contribution of any single variable to the corrected score, the choice of weights depends on the relative importance of Y_0_ and Z_0_ in the specific context of analysis. For instance, if the physical constrain is considered more critical than the environmental limitation, its weight might be set higher than that of the other variable. So, we propose adjustments with levels of weight disparity in relation to ability and opportunity: null weight differences (w_y_ = w_z_ = 0.5), moderate differences (w_y_ = 0.6, w_z_ = 0.4 or w_y_= 0.7, w_z_=0.3), or large differences (w_y_ = 0.8, w_z_ = 0.2 or w_y_ = 0.9, w_z_ = 0.1). The clinician freely can choose the weight to assign to the variables following their personal beliefs on the matter, based on their clinical experience or research findings. This weighting approach aligns with standard practices in statistical modelling, where weights are allocated proportionally to the expected significance of each variable (for a detailed discussion on weight allocation strategies in predictive models [26]). This flexibility allows researchers to adapt the model to the specific characteristics of the population or contextual, or cultural factors, while maintaining the interpretability and consistency of the scoring framework.

Once the adjustment factor is calculated, it is normalized using an exponential function, which converts the raw differences into a proportion that can be applied to the score

2 $$\:Normalized\:adjustment = \frac{{{\text{exp}}\left( {\:{w_{y\:}}.\left( {Y - {X_{O\:}}} \right) + {w_{Z\:}}.\left( {Z - {Z_{O\:}}} \right)} \right) - 1}}{{{\text{exp}}\left( k \right) - 1}}$$

where *k* is the scaling constant that adjusts the magnitude of the adjustment and normalizes it within the desired range. The scaling constant k is calculated based on the expected maximum deviation, ensuring that the correction does not exceed a reasonable range. We can assume *k* equal to 3 because it represents the maximum weighted deviation, ensuring the exponential correction scales appropriately within the predefined range of the variables. The adjustment is applied in a subtractive manner, meaning the calculated adjustment is subtracted from the original score. This ensures that the score is not artificially inflated but can still decrease based on the limiting variables.

The final corrected score is calculated as:

3 $$\:{\:{\:X}_{c\:}=X}_{O\:}-\text{m}\text{i}\text{n}(\Delta\:,\text{max}\left(0,\:Normalized\:adjustment\right)$$

where Δ is the maximum allowable deviation. This step ensures that the adjustment is bounded, and the score does not vary excessively. The adjustment factor is restricted to a value within the defined bounds, such as Δ = 1, meaning that no correction will result in a score change larger than 1 point, ensuring the result stays within the established range. The result is a corrected score of frequency of activity for each item that incorporates the influence of the limiting variables while maintaining the integrity of the original score and its range. This controlled correction process allows for small, context-specific changes that reflect real-world conditions without drastic shifts in the overall score. Considering the above, the total UJACAS score will also change from 272 (i.e. 17 items x 4 = max score x 4 variables) to 68 (i.e. 17 items on corrected activity frequency x 4 = max score).

The described approaches are consistent with the principles of adaptive measurement in disability studies [27] and would provide a more nuanced and favourable evaluation of AA in case of disabilities and/or environmental obstacles. The methodology presented leverages a weighted exponential adjustment to correct an individual’s score on a specific item based on the influence of barriers, such as ability to act and opportunity to act. The core rationale behind this approach is grounded in the concept of true score estimation, where the objective is to adjust the observed score while maintaining the underlying range of values. This is done by calculating a correction factor, which is then applied to the original score in a subtracting manner to ensure the correction remains within a reasonable bound. The correction factor is derived from an exponential function, reflecting how the deviation of ability to act and opportunity to act influences the target score. To avoid excessive shifts in the final score for the single item, a scaling constant and a maximum allowable deviation are introduced to constrain the magnitude of adjustment, ensuring that the corrected score stays within the established range.

To assess discrepancies between the score X_0_ and its prerequisite variables Y_0_ and Z_0_, we opted to calculate Y_0_ - X_0_ and Z_0_ - X_0_. This approach allows us to measure how far the observed outcome X_0_ deviates from the expected contributions of Y_0_ and Z_0_ individually, offering a clearer understanding of specific discrepancies. This method emphasizes the individual contributions of Y_0_ and Z_0_ to X_0_, avoiding the potential interpretative challenges of combining the variables into a single aggregate discrepancy as in X_0_ - Y_0_ - Z_0_. It is particularly suitable when the focus is on isolating specific shortfalls rather than evaluating a combined shortfall.

This method is particularly useful when the goal is to minimize drastic changes while allowing small, context-specific adjustments, reflecting real-world conditions where scores should not fluctuate excessively. Moreover, the introduction of a precondition—where any zero input automatically results in a zero output—is essential because zero scores cannot be effectively weighted using the formula. This exclusion is a necessary constraint, albeit one that reduces the generalizability of the formula. Furthermore, the weight assigned to ability to act is intended to reflect how much an individual’s perceived capability or skill in performing the activity influences their actual participation; and the weight assigned to opportunity may be lower than that for ability, reflecting the fact that while opportunity can enhance participation, it is less influential than personal capability when constraints are severe. Thus, the approach provides a balanced and controlled way to adjust scores without distorting the results or causing over-corrections.This model could first estimate the impact that (the reduction in) ability and (the lack of) opportunities has on limiting each specific type of activity. For example, an individual with arthritis might engage in fewer physical activities, but the severity of their condition should be considered when adjusting their score. They would then proceed to aggregate the corrected frequencies of activities based on the level of impairment, considering both personal and environmental limitations.

The table of correction, based on the above mentioned equation, and two vignettes regarding the comparison of two ideal people evaluated through the UJACAS, are provided in the supplementary materials (S1).

## Discussion

The UJACAS stands out among other AA measurement tools due to its multidimensional approach, which incorporates not only the frequency of activity but also motivation, ability, and opportunities to engage. In contrast, other commonly used tools like the World Health Organization’s Active Ageing Index (AAI) primarily focus on macro-level indicators like health and socioeconomic factors [1]. While these tools provide valuable insights, they tend to overlook individual-level factors such as personal barriers and motivations, which are crucial for a nuanced understanding of active ageing. The UJACAS’s inclusion of these dimensions allows for a more tailored evaluation that considers both the external and internal factors affecting engagement. However, a limitation of UJACAS is that its scoring model may not be universally applicable across different cultural or environmental contexts, which necessitates adaptation. The refined UJACAS model, with its adjustments for personal and environmental barriers, has significant implications for research and practice. In clinical settings, it can provide a more accurate assessment of an individual’s capacity for active ageing, helping healthcare professionals design interventions that account for both intrinsic and extrinsic factors influencing activity levels. Public health programs can use the tool to identify subgroups of older adults who may benefit from targeted interventions that address specific barriers, such as limited mobility or lack of social engagement. In community programs, the model’s focus on motivation and opportunity can guide the development of more inclusive and accessible initiatives that encourage participation from diverse groups, including those with disabilities or chronic conditions. By tailoring interventions to the specific needs of individuals, the UJACAS model can improve the overall effectiveness of active ageing initiatives.

Future research on AA would benefit from integrating biological, psychological, and social factors to provide a more holistic understanding of ageing. For example, combining biological markers, such as telomere length or epigenetic changes, with psychosocial factors could offer a comprehensive assessment of an individual’s capacity for active ageing. Additionally, understanding how resilience and coping mechanisms interact with these biological factors can help identify strategies to mitigate the impact of age-related decline. Interdisciplinary collaboration across fields like gerontology, psychology - and in particular *geropsychology*, an ever-growing approach - public health, and biology will be essential in advancing our understanding of active ageing and improving interventions for older adults [28–29].

## Conclusion

In conclusion, AA concept can navigate and overcome personal and environmental limitations, and it is not merely the proxy of how often older adults participate in activities. Future assessments should consider the interplay between activity levels and the barriers faced, ensuring that those who remain engaged despite adversity are recognized for their resilience. By addressing personal limitations and environmental obstacles, the refined model of AA enables more accurate evaluations and, consequently, more effective interventions.

Standardizing UJACAS adaptations across different cultural and environmental contexts is essential to enhance the comparability of results and ensure that the tool can be effectively used in diverse populations. This process will help eliminate inconsistencies in scoring and interpretation, making it a more reliable instrument for assessing Active Ageing, globally. By establishing clear guidelines for adaptation and the scoring computation, UJACAS can maintain its robustness while being tailored to the specific needs of various regions, facilitating cross-national research and comparative studies.

Finally, addressing AA as a political and social issue involves ensuring that opportunities for health and well-being are not determined by socioeconomic status. By focusing on equity, inclusivity, and tailored interventions, policies and programs can promote Active Ageing without forgetting that limitations exist in ageing, and that the level of activity must be adapted to the individual’s actual conditions. The refined AA model supports the development of equitable health and ageing policies by recognizing the diverse challenges faced by older adults and providing a framework for addressing them.

## Electronic supplementary material

Below is the link to the electronic supplementary material.

Supplementary Material 1

## Data Availability

No datasets were generated or analysed during the current study.
