# Supplementary material for: Navigating the maze of active ageing measurement: untangling methodological and theoretical issues in the UJACAS questionnaire
Source: Aging Clin Exp Res. 2025 Mar 13;37(1):83. doi: 10.1007/s40520-025-02953-5 (PMC11903560; doi:10.1007/s40520-025-02953-5)
Supplement: Supplementary file 1 — Supplementary Material 1 [file 40520_2025_2953_MOESM1_ESM.docx]

|  |  |  |  |  |  |  |  |  |  |
| --- | --- | --- | --- | --- | --- | --- | --- | --- | --- |
| X_o_ | Y_o_ | Z_o_ |  | X_c1_ | X_c2_ | X_c3_ | X_c4_ | X_c5_ |  |
|  |  |  |  |  |  |  |  |  |  |
| 0 or | 0 or | 0 |  | 0.000 | 0.000 | 0.000 | 0.000 | 0.000 |  |
| 1 | 4 | 4 |  | 0.000 | 0.000 | 0.000 | 0.000 | 0.000 |  |
| 1 | 3 | 4 |  | 0.414 | 0.475 | 0.530 | 0.580 | 0.625 |  |
| 1 | 4 | 3 |  | 0.414 | 0.347 | 0.273 | 0.191 | 0.100 |  |
| 1 | 2 | 4 |  | 0.665 | 0.735 | 0.793 | 0.840 | 0.878 |  |
| 1 | 4 | 2 |  | 0.665 | 0.580 | 0.475 | 0.347 | 0.191 |  |
| 1 | 3 | 3 |  | 0.665 | 0.665 | 0.665 | 0.665 | 0.665 |  |
| 1 | 1 | 4 |  | 0.818 | 0.878 | 0.924 | 0.957 | 0.982 |  |
| 1 | 4 | 1 |  | 0.818 | 0.735 | 0.625 | 0.475 | 0.273 |  |
| 1 | 2 | 3 |  | 0.818 | 0.840 | 0.860 | 0.878 | 0.895 |  |
| 1 | 3 | 2 |  | 0.818 | 0.793 | 0.766 | 0.735 | 0.702 |  |
| 1 | 1 | 3 |  | 0.910 | 0.936 | 0.957 | 0.974 | 0.988 |  |
| 1 | 3 | 1 |  | 0.910 | 0.878 | 0.840 | 0.793 | 0.735 |  |
| 1 | 2 | 2 |  | 0.910 | 0.910 | 0.910 | 0.910 | 0.910 |  |
| 1 | 1 | 2 |  | 0.966 | 0.974 | 0.982 | 0.988 | 0.994 |  |
| 1 | 2 | 1 |  | 0.966 | 0.957 | 0.947 | 0.936 | 0.924 |  |
| 1 | 1 | 1 |  | 1.000 | 1.000 | 1.000 | 1.000 | 1.000 |  |
| 2 | 4 | 4 |  | 1.665 | 1.665 | 1.665 | 1.665 | 1.665 |  |
| 2 | 3 | 4 |  | 1.818 | 1.840 | 1.860 | 1.878 | 1.895 |  |
| 2 | 4 | 3 |  | 1.818 | 1.793 | 1.766 | 1.735 | 1.702 |  |
| 2 | 2 | 4 |  | 1.910 | 1.936 | 1.957 | 1.974 | 1.988 |  |
| 2 | 4 | 2 |  | 1.910 | 1.878 | 1.840 | 1.793 | 1.735 |  |
| 2 | 3 | 3 |  | 1.910 | 1.910 | 1.910 | 1.910 | 1.910 |  |
| 2 | 1 | 4 |  | 1.966 | 1.988 | 2.000 | 2.000 | 2.000 |  |
| 2 | 4 | 1 |  | 1.966 | 1.936 | 1.895 | 1.840 | 1.766 |  |
| 2 | 2 | 3 |  | 1.966 | 1.974 | 1.982 | 1.988 | 1.994 |  |
| 2 | 3 | 2 |  | 1.966 | 1.957 | 1.947 | 1.936 | 1.924 |  |
| 2 | 1 | 1 |  | 2.000 | 2.000 | 2.000 | 2.000 | 2.000 |  |
| 2 | 1 | 2 |  | 2.000 | 2.000 | 2.000 | 2.000 | 2.000 |  |
| 2 | 2 | 1 |  | 2.000 | 2.000 | 2.000 | 2.000 | 2.000 |  |
| 2 | 1 | 3 |  | 2.000 | 2.000 | 2.000 | 2.000 | 2.000 |  |
| 2 | 3 | 1 |  | 2.000 | 1.988 | 1.974 | 1.957 | 1.936 |  |
| 2 | 2 | 2 |  | 2.000 | 2.000 | 2.000 | 2.000 | 2.000 |  |
| 3 | 4 | 4 |  | 2.910 | 2.910 | 2.910 | 2.910 | 2.910 |  |
| 3 | 3 | 4 |  | 2.966 | 2.974 | 2.982 | 2.988 | 2.994 |  |
| 3 | 4 | 3 |  | 2.966 | 2.957 | 2.947 | 2.936 | 2.924 |  |
| 3 | 1 | 1 |  | 3.000 | 3.000 | 3.000 | 3.000 | 3.000 |  |
| 3 | 1 | 2 |  | 3.000 | 3.000 | 3.000 | 3.000 | 3.000 |  |
| 3 | 2 | 1 |  | 3.000 | 3.000 | 3.000 | 3.000 | 3.000 |  |
| 3 | 1 | 3 |  | 3.000 | 3.000 | 3.000 | 3.000 | 3.000 |  |
| 3 | 3 | 1 |  | 3.000 | 3.000 | 3.000 | 3.000 | 3.000 |  |
| 3 | 1 | 4 |  | 3.000 | 3.000 | 3.000 | 3.000 | 3.000 |  |
| 3 | 2 | 2 |  | 3.000 | 3.000 | 3.000 | 3.000 | 3.000 |  |
| 3 | 4 | 1 |  | 3.000 | 3.000 | 2.994 | 2.974 | 2.947 |  |
| 3 | 2 | 3 |  | 3.000 | 3.000 | 3.000 | 3.000 | 3.000 |  |
| 3 | 3 | 2 |  | 3.000 | 3.000 | 3.000 | 3.000 | 3.000 |  |
| 3 | 2 | 4 |  | 3.000 | 3.000 | 3.000 | 3.000 | 3.000 |  |
| 3 | 4 | 2 |  | 3.000 | 2.988 | 2.974 | 2.957 | 2.936 |  |
| 3 | 3 | 3 |  | 3.000 | 3.000 | 3.000 | 3.000 | 3.000 |  |
| 4 | Any Y_o_ | Any Z_o_ |  | 4.000 | 4.000 | 4.000 | 4.000 | 4.000 |  |
|  |  |  |  |  |  |  |  |  |  |

X_o_, Y_o_, and Z_o_ represent the observed scores for frequency of activity, ability to act, and opportunity to act. respectively. From X_c1_ to X_c5_ are the corrected scores based on the correction equation described in the paper, corresponding to the different corrections obtained by varying the weights w_y_ and w_z_ assigned to variables Y and Z. Specifically, for X_c1_, both weights are set to 0.5; for _Xc2_, w_y_ =0.6 and w_z_ =0.4; for X_c3_, w_y_ =0.7 and w_z_ =0.3; for X_c4_, w_y_ =0.8 and w_z_ =0.2; and for X_c5_, w_y_ =0.9 and w_z_ =0.1. The different weights reflect the degree of importance assigned to ability to act and opportunity to act in promoting / constrain the frequency of activity. Indeed, there are reasons to assume that the weight of abilities (or conversely, personal constraints) should be considered greater than the weight of environmental constraints. Alternatively, X_c1_ can be used, as it considers the two dimensions to have equivalent weight in determining activity frequency.

The following are two vignettes regarding the comparison of two ideal people evaluated through the UJACAS with the original and the proposed methodology.

**Vignette 1: Mr. Antonio F., 76 Years Old**. Mr. Antonio, a 76-year-old man, lives on the outskirts of town, far from the city center. He has been dealing with arthritis for several years, which significantly affects his mobility. His physical limitations make it challenging to move around, especially to participate in activities that require him to travel long distances. On top of his health concerns, his residence in a peripheral area limits his access to public transportation and community services. Despite these challenges, Mr. Antonio remains engaged in social activities, contributing to his community through volunteering and supporting local initiatives. On the item *I contribute to activities that benefit the community*, he rated his will to act “4”, his activity as a “3” indicating moderate/large engagement. However, when asked about his ability to act and opportunity to act, he rated both as “2” reflecting the substantial barriers he faces due to his physical and environmental limitations. His final score was: 11/16 (69% of total score). According to the correction equation proposed by the present study Mr. Antonio obtained a corrected score of 3/4 (75% of the maximum score).

**Vignette 2: Mr. Giuseppe L., 74 Years Old**.

Mr. Giuseppe, a 74-year-old man, lives in a downtown apartment close to shops, public transportation, and various community facilities. He does not suffer from any significant physical ailments and reports being in good health. His central location allows him to easily access and participate in a variety of community activities, and he is highly involved in social engagements, such as volunteering and supporting local events. On the item *I contribute to activities that benefit the community*, he rated he rated his will to act “4”, his activity as a “3” indicating moderate/large engagement showing similar engagement to Mr. Antonio. However, he rated his ability to act and opportunity to act as “4” indicating that his health and convenient living situation greatly facilitate his active participation. His final score was: 15/16 (94% of the maximum score). The corrected score of Mr. Giuseppe becomes 2.910/4 (73% of the maximum score).

The UJACAS scoring system's traditional approach can partially misrepresent participation. Giuseppe may appear significantly more active than Antonio, but he merely benefits from more favorable internal and external conditions. The model presented here aims to amend this bias by correcting activity frequency based on the varying levels of difficulty people encounter, leading to a more accurate and fair evaluation.
